# Sex-based associations with microvascular injury and outcomes after ST-segment elevation myocardial infarction

**DOI:** 10.1136/openhrt-2018-000979

**Published:** 2019-04-29

**Authors:** Annette Marie Maznyczka, David Carrick, Jaclyn Carberry, Kenneth Mangion, Margaret McEntegart, Mark C Petrie, Hany Eteiba, Mitchell Lindsay, Stuart Hood, Stuart Watkins, Andrew Davie, Ahmed Mahrous, Ian Ford, Paul Welsh, Naveed Sattar, Keith G Oldroyd, Colin Berry

**Affiliations:** 3 Robertson Centre for Biostatistics, University of Glasgow, Glasgow, UK; 1 British Heart Foundation Glasgow Cardiovascular Research Centre, Institute of Cardiovascular and Medical Sciences, University of Glasgow, Glasgow, UK; 2 West of Scotland Heart and Lung Centre, Golden Jubilee National Hospital, Glasgow, UK; 3 Robertson Centre for Biostatistics, University of Glasgow, Glasgow, UK

**Keywords:** sex, myocardial infarction, clinical outcomes, index of microcirculatory resistance, microvascular obstruction, MRI

## Abstract

**Objectives:**

We aimed to assess for sex differences in invasive parameters of acute microvascular reperfusion injury and infarct characteristics on cardiac MRI after ST-segment elevation myocardial infarction (STEMI).

**Methods:**

Patients with STEMI undergoing emergency percutaneous coronary intervention (PCI) were prospectively enrolled. Index of microcirculatory resistance (IMR) and coronary flow reserve (CFR) were measured in the culprit artery post-PCI. Contrast-enhanced MRI was used to assess infarct characteristics, microvascular obstruction and myocardial haemorrhage, 2 days and 6 months post-STEMI. Prespecified outcomes were as follows: (i) all-cause death/first heart failure hospitalisation and (ii) cardiac death/non-fatal myocardial infarction/urgent coronary revascularisation (major adverse cardiovascular event, MACE) during 5- year median follow-up.

**Results:**

In 324 patients with STEMI (87 women, mean age: 61 ± 12.19 years; 237 men, mean age: 59 ± 11.17 years), women had anterior STEMI less often, fewer prescriptions of beta-blockers at discharge and higher baseline N-terminal pro-B-type natriuretic peptide levels (all p < 0.05). Following emergency PCI, fewer women than men had Thrombolysis in Myocardial Infarction (TIMI) myocardial perfusion grades ≤ 1 (20% vs 32%, p = 0.027) and women had lower corrected TIMI frame counts (12.94 vs 17.65, p = 0.003). However, IMR, CFR, microvascular obstruction, myocardial haemorrhage, infarct size, myocardial salvage index, left ventricular remodelling and ejection fraction did not differ significantly between sexes. Female sex was not associated with MACE or all-cause death/first heart failure hospitalisation.

**Conclusion:**

There were no sex differences in microvascular pathology in patients with acute STEMI. Women had less anterior infarcts than men, and beta-blocker therapy at discharge was prescribed less often in women.

**Trial registration number:**

NCT02072850.

**Key questions**

**What is already know about the subject?**

- Women with ST-segment elevation myocardial infarction (STEMI) have reportedly worse outcomes than men and microvascular pathology has been postulated as a potential mechanism.
- Findings from non-invasive imaging studies are conflicting, some report smaller infarcts in women while others report no sex differences.
- Previous studies did not use MRI methods to detect myocardial haemorrhage (microvascular destruction) and most acquired MRI at a single time point.

**What does this study add?**

- There were no sex differences in acute microvascular reperfusion injury with index of microcirculatory resistance, or on MRI.
- Women had fewer anterior myocardial infarcts and were prescribed beta-blockers at discharge less often than men.

**How might this impact on clinical practice?**

- The hypothesis of sex differences in acute microvascular injury for STEMI is not supported by this study.
- This study serves a reminder of sex differences in post-MI care in contemporary practice, and the need to reduce sex imbalance in management.

## Introduction

Ischaemic heart disease is the leading cause of death and disability worldwide.^1^ Although many studies have reported worse outcomes in women after ST-segment elevation myocardial infarction (STEMI),^2 3^ the results are conflicting.^4 5^

Confounders, including older age^6^ and comorbidities,^7^ particularly diabetes mellitus,^8^ and renal insufficiency,^3 6^ may contribute to excess mortality in women post-STEMI. Another confounder is longer symptom to reperfusion times in women^6 8–10^ purportedly attributable to women underestimating their cardiovascular risk or misinterpreting the symptoms which may be atypical in nature.^11^ Sex disparity in guideline-directed pharmacological^2^ and invasive reperfusion treatments has also been reported.^3 10–12^ Reducing sex imbalance in management and outcomes post-STEMI, and identifying potential mechanistic explanations, is emphasised in guideline recommendations.^2 13^

Findings from previous studies on sex and infarct size assessed by MRI are conflicting; some report smaller infarct size and greater myocardial salvage in women^4^ while others reported no sex differences.^6–8 14^ A previous study using single-photon emission CT also observed better myocardial salvage after primary percutaneous coronary intervention (PCI) in women.^5^ Limitations of these studies include not using specific MRI methods to detect myocardial haemorrhage (a consequence of severe microcirculatory injury) and the acquisition of MRI at a single early time point post-STEMI in most,^6–8 14^ but not all, studies.^4^ This is relevant since the size of infarction evolves dynamically post-STEMI. Furthermore, patients included in some studies were pooled from multiple randomised trials.^5 8^

Microvascular dysfunction has been postulated as a potential mechanism for worse outcomes in women.^8^ We investigated sex associations with the incidence, nature and timecourse of reperfusion injury in patients after an acute STEMI using invasive measures of microvascular function acutely, and serial assessments with multiparametric contrast-enhanced MRI.^15^ We also aimed to assess the prognostic implications on left ventricular (LV) surrogate outcomes revealed by MRI at 6 months and health outcomes in the longer term.

## Methods

### Study population

We performed a retrospective analysis of a prospectively collected cohort of patients with acute STEMI in a regional cardiac centre from July 2011 to November 2012. Consecutive, unselected patients with STEMI were screened for inclusion in the study and patients with MRI contraindications were excluded. The study was approved by the National Research Ethics Service (reference 10-S0703-28).

Sociodemographic status was identified from postcodes at the time of enrolment into the study, using the Scottish Index of Multiple Deprivation (SIMD) tool. The first and fifth SIMD quintiles contain the 20% most deprived and least deprived postal zones in Scotland, respectively.

### Invasive physiology, angiographic and ECG measures of microvascular injury

Index of microcirculatory resistance (IMR) was measured in the culprit coronary artery using a coronary guidewire with a pressure sensor and temperature sensor (Abbott Vascular, Santa Clara, CA, USA) at the end of primary PCI or rescue PCI, as previously described.^15^ IMR is defined as distal coronary pressure multiplied by mean transit time of a bolus of saline at room temperature, during maximal coronary hyperaemia (induced by 140 µg/kg/min of intravenous adenosine preceded by an intracoronary bolus of 200 µg of nitrate).^15^ Coronary flow reserve (CFR) is the mean transit time at rest divided by the mean transit time during hyperaemia.

Thrombolysis in Myocardial Infarction (TIMI) myocardial perfusion grade and corrected TIMI frame count were evaluated at the end of the PCI procedure. If the infarct-related vessel was the left anterior descending artery (LAD), the frame count was divided by 1.7 to correct for longer vessel length. Complex plaques were defined as lesions ≥30% diameter stenosis with ≥2 adverse features on a five-point plaque characterisation score comprising the following: presence of a filling defect/thrombus; ulceration; irregularity; TIMI flow <3, moderate/severe calcification or involvement of a bifurcation.^16^ Intraprocedural thrombotic events were defined as new or increasing thrombus, abrupt vessel closure, no reflow or slow reflow, or distal embolisation at any time during PCI.^17^ ST-segment resolution was assessed 60 min after reperfusion and was compared with ECGs obtained before coronary reperfusion.

### Cardiac MRI

**Figure 1:**
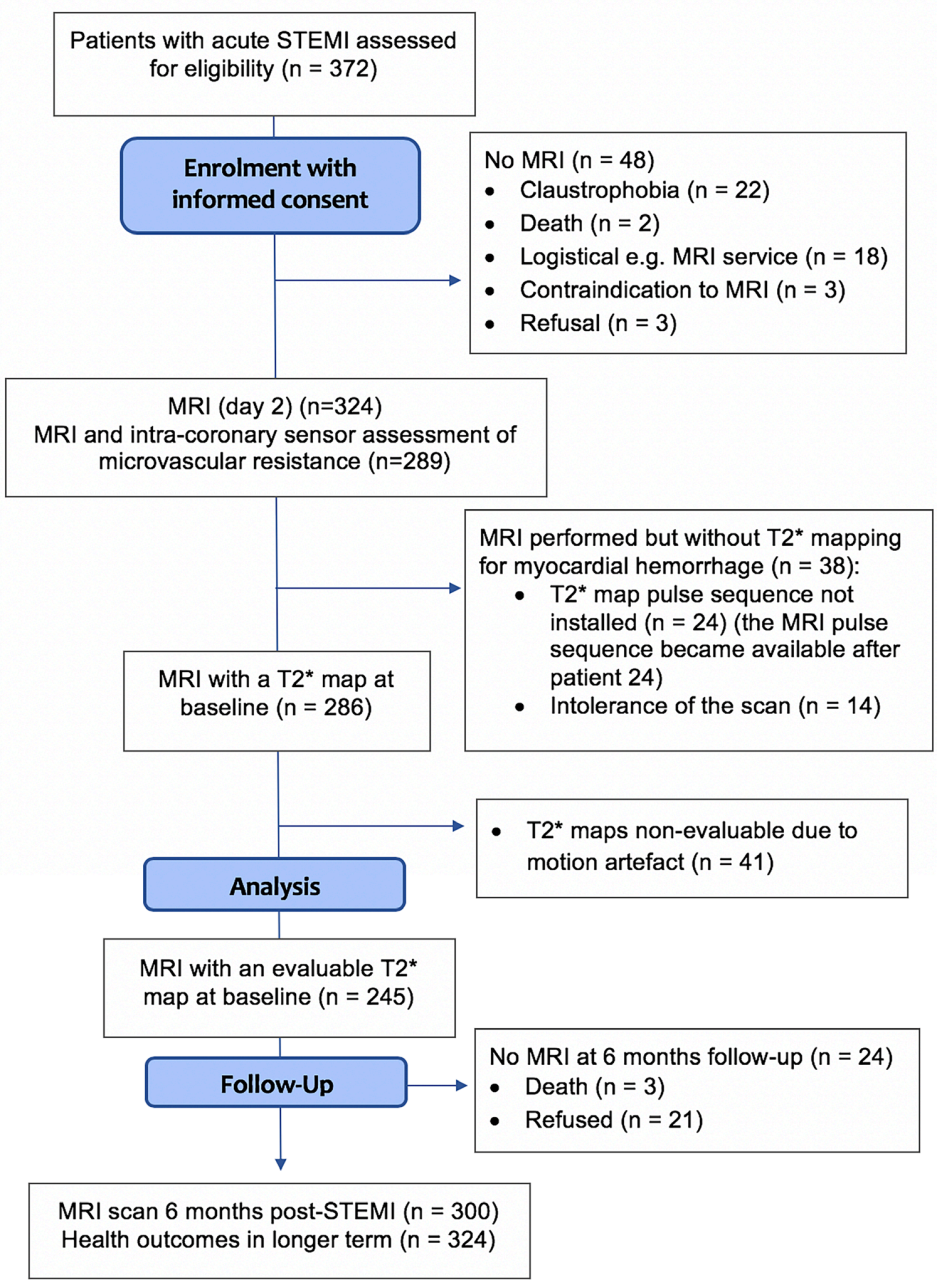
Consort flow diagram of the study. STEMI, ST-segment elevation myocardial infarction.

MRI was performed on a Siemens MAGNETOM Avanto (Erlangen, Germany) 1.5-Tesla scanner at 2 days post-STEMI and 6 months later (figure 1). The imaging protocol (Supplementary Methods) included cine MRI with steady-state free precession, T2-mapping,^18^ native T1 mapping,^19^ T2*-mapping^14^ and delayed-enhancement phase-sensitive inversion-recovery pulse sequences.^20^ The scan acquisitions were spatially co-registered and included different slice orientations to enhance diagnostic confidence.

### Image analyses

The MRI analyses are described in detail in the Supplementary Methods. The results of infarct characteristics are reported for the whole of the LV.

#### Infarct size, microvascular obstruction and myocardial haemorrhage

The presence of acute infarction was established based on abnormalities in cine wall motion, rest first-pass myocardial perfusion and delayed-enhancement imaging in two imaging planes. The myocardial mass of late gadolinium was quantified using computer-assisted planimetry and the territory of infarction was delineated using a signal intensity threshold of >5 standard deviations (SDs above a remote reference region and expressed as a percentage of total LV mass.^14^ Late gadolinium enhancement and microvascular obstruction were expressed as % LV mass.

Microvascular obstruction was defined as a dark zone on late gadolinium enhancement imaging at least 1 min post-contrast injection that persisted within an area of late gadolinium enhancement at 15 min. On the T2* maps, a region of reduced signal intensity within the infarcted area, with a T2* value of <20 ms^14^ was considered to confirm the presence of myocardial haemorrhage.

#### Myocardial salvage

Myocardial salvage was calculated by subtraction of the infarct size at 6 months from the baseline area at risk.^14^ The myocardial salvage index was calculated by dividing the myocardial salvage area by the initial area at risk.

#### LV remodelling

An increase in ≥20% LV end-diastolic volume (LVEDV) at 6 months from baseline was taken to reflect adverse LV remodelling.^19^

### Clinical outcomes

The prespecified primary composite outcome was all-cause death or the first heart failure hospitalisation following the initial admission. The second composite outcome was major adverse cardiovascular events (MACE) including cardiac death, non-fatal myocardial infarction (MI) or urgent coronary revascularisation (Supplementary Methods).

#### Statistical analyses

Statistical analyses were performed using SPSS (V.24.0, SPSS, IBM, Armonk, NY, USA) and MedCalc Statistical Software V.18 (MedCalc Software, Ostend, Belgium). Continuous variables were tested for normality using the Shapiro-Wilk test, and were presented as mean±SD, or median and interquartile range (IQR) as appropriate. Continuous and categorical between-group comparisons were based on χ^2^ and t-tests, respectively, unless indicated otherwise (tables 1–3). Associations between baseline covariates and MRI outcomes were evaluated using linear or logistic regression for continuous or categorical outcomes, respectively. Associations between baseline covariates and clinical outcomes were evaluated using cox proportional hazards analyses. Non-sex (woman/ man) variables that were significant (p<0.05) were included in multivariable analyses to assess for associations between sex and parameters from the angiographic, ECG and MRI analyses. Associations were expressed as odds ratios (OR), or hazard ratios (HR), with 95% confidence intervals (CIs). Predictors of late mortality (median 5-year follow-up) were assessed only in those patients who survived to hospital discharge (landmark survival analysis). Kaplan-Meier survival plots were constructed for visualisation and assessed using the log-rank test.

**Table 1:** Baseline characteristics

Characteristics*	Women (n=87)	Men (n=237)	P value
Age (years), mean±SD	61.18±12.19	58.61±11.17	0.074*
Current smoker, n (%) BMI (kg/m^2^), median (IQR)	57 (66) 28.50 (25.10–32.30)	139 (59) 28.30 (25.78–31.20)	0.262† 0.664‡
BMI <25 (kg/m^2^), n (%) 25≤BMI<30 (kg/m^2^), n (%) BMI≥30 (kg/m^2^), n (%)	18 (21) 29 (33) 40 (46)	44 (19) 112 (47) 81 (34)	0.069†
Hypertension, n (%)	32 (37)	73 (31)	0.308†
Hypercholesterolaemia, n (%)	28 (32)	66 (28)	0.446†
Diabetes mellitus, n (%)	8 (9)	26 (11)	0.644†
Previous MI, n (%)	5 (6)	20 (8)	0.421†
Previous PCI, n (%)	2 (2)	16 (7)	0.171§
Pre-infarct angina, n (%)	9 (10)	31 (13)	0.507†
Social deprivation status, n (%):			
I II III IV V	31 (36) 16 (19) 16 (19) 11 (13) 12 (14)	81 (35) 53 (23) 38 (16) 39 (17) 23 (10)	0.750†
Presenting HR (bpm), median (IQR)	80.00 (67.50–91.00)	76.00 (65.00–87.00)	0.092‡
Presenting BP <90/60 (mm Hg), n (%)	14 (16)	21 (9)	0.063†
Ventricular fibrillation/tachycardia, n (%)	8 (9)	14 (6)	0.297†
Symptom to reperfusion time (min), median (IQR)	172.50 (122.00–316.00)	174.00 (119.00–314.00)	0.746‡
Symptom to reperfusion time >6 hours, n (%)	16 (20)	41 (18)	0.840†
Door-to-balloon time (min), median (IQR)	20.00 (16.00–23.00)	19.00 (16.00–24.00)	0.477‡
Killip class, n (%):			
I II III/IV	65 (75) 15 (17) 7 (8)	168(71) 53 (22) 16 (7)	0.584†
Reperfusion strategy, n (%):			0.458§
Primary PCI	83 (95)	219 (92)	
Thrombolysis (failed/successful)	4 (5.2)	18 (8.8)	
Aspiration thrombectomy, n (%)	57 (66)	179 (76)	0.073†
Glycoprotein IIbIIIa inhibitor, n (%)	82 (94)	215 (91)	0.307†
***Medications at discharge:***			
ACE-I or ARB, n (%)	85 (98)	235 (99)	0.293†
Beta-blocker, n (%)	79 (91)	229 (97)	**0.032**†
Statin, n (%)	87 (100)	237 (100)	
Aspirin, n (%)	86 (99)	237 (100)	0.098†
Clopidogrel, n (%) Ticagrelor, n (%)	86 (99) 1 (1)	235 (99) 2 (1)	0.799†
***Blood results on admission:***			
Anaemia, n (%)	17 (20)	41 (17)	0.641†
eGFR ≥60 (mL/min/1.73 m^2^), n (%) 30≤eGFR<60 (mL/min/1.73 m^2^), n (%) eGFR <30 (mL/min/1.73 m^2^), n (%)	76 (87) 11 (13) 0	222 (94) 14 (6) 0	0.059†
C-reactive protein, mg/L, median (IQR)	4.00 (2.00–9.00)	3.00 (2.00–7.00)	0.063‡
Peak troponin T, ng/L, median (IQR)	1334.00 (83.60–3548.00)	1944.50 (165.40–5437.50)	0.113‡
N-terminal pro-B-type natriuretic peptide, median (IQR)	1175.00 (627.50–2285.50)	701.50 (309.00–1456.00)	**0.009**‡
***Blood results at 6 months post-STEMI***			
N-terminal pro-B-type natriuretic peptide, median (IQR)	230.50 (97.00–510.50)	154.00 (73.00–337.00)	0.144‡

Presenting HR was available in 321 subjects. Symptom-to-reperfusion time was available in 304 subjects. Door-to-balloon time was available in 305 subjects. eGFR was available in 323 subjects. C-reactive protein was available in 316 subjects. Peak troponin was available in 313 subjects. N-terminal pro-B-type natriuretic peptide results were available in 139 subjects on admission and 171 subjects at 6 months. Results of interleukin 6 were available in 139 subjects on admission and 171 subjects at 6 months. Information on anaemia was available in 324 subjects and was defined as haemoglobin<130 g/L in men or <115 g/L in women.

*Student’s t-test.

†χ^2^ test.

‡Mann-Whitney U test.

§Fisher’s exact test.

ACE-I, ACE inhibitor; ARB, angiotensin receptor blocker; BMI, body mass index; BP, blood pressure; eGFR, estimated glomerular filtration rate; HR, heart rate; MI, myocardial infarction; PCI, percutaneous coronary intervention; STEMI, ST-segment elevation myocardial infarction.

**Table 2:** ECG, invasive coronary physiology and angiographic characteristics

	Women (n=87)	Men (n=237)	P value
**Angiography:**			
Number of diseased coronary arteries, n (%):			
1 2 3	48 (55) 27 (31) 12 (14)	126 (53) 72 (30) 39 (17)	0.842*
Culprit artery, n (%): LAD or left main Right coronary artery Circumflex	27 (31)/0 50 (58) 10 (12)	93 (39)/1 (0) 94 (39.7) 49 (21)	0.013*
TIMI flow grade pre-PCI ≤1, n (%)	65 (75)	162 (68)	0.268*
TIMI flow grade post-PCI ≤2, n (%)	4 (5)	19 (8)	0.288*
TIMI frame count post-PCI, median (IQR)	12.94 (10.00–18.62)	17.65 (10.00–27.77)	**0.003†**
TIMI myocardial perfusion grade post-PCI, n (%): ≤1 ≥2	17 (20) 70 (81)	76 (32) 161 (68)	0.027*
**Plaque characteristics:**			
Culprit lesion plaque characterisation score, n (%): ≤Median (4) >Median (4)	76 (87) 11 (13)	193 (81) 44 (19)	0.208*
Number of complex plaques in culprit/non-culprit coronary arteries, n (%): ≤1 ≥2	65 (75) 22 (25)	180 (76) 57 (24)	0.818*
Intraprocedural thrombotic event occurrence, n (%)	8 (9)	25 (11)	0.721*
**Invasive coronary physiology:**			
IMR (U), median (IQR)	23.10 (15.00–41.00)	25.50 (15.33–47.68)	0.199†
IMR >40(U), n (%)	21 (27)	60 (28)	0.771*
CFR, median (IQR)	1.50 (1.10–2.03)	1.60 (1.10–2.10)	0.427†
CFR≤2, n (%)	13 (17)	37 (18)	0.871*
**ECG:**			
ST-segment resolution, n (%): Complete, ≥70% Incomplete, 30% to <70% None, ≤30%	44 (51) 31 (36) 12 (14)	104 (44) 96 (41) 36 (15)	0.580*

TIMI frame count post-PCI was available in 322 subjects. IMR was available in 289 subjects. CFR was available in 286 subjects. Information on ST-segment resolution was available in 323 subjects.

*χ^2^Chi-square test.

†Mann-Whitney U test.

CFR, coronary flow reserve; IMR, Index of microcirculatory resistance; LAD, left anterior descending artery; PCI, percutaneous coronary intervention; TIMI, Thrombolysis in Myocardial Infarction.

**Table 3:** Cardiac MRI findings at 2 days and 6 months post-reperfusion in patients with STEMI

	Female (n=87)	Male (n=237)	P value
MRI finding 2 days post-MI			
Infarct size (%LV mass), median (IQR) LVEF%, mean±SD	15.90 [4.90–24.28) 56.5±9.8	16.50 [7.00–28.88) 53.2±9.5	0.141* 0.086†
LVESVi, mL/m^2^, median (IQR)	32.45 [23.40–40.00)	36.30 [27.40–46.30)	< 0.0001*
LVEDVi, mL/m^2^, median (IQR)	70.80 [63.70–80.60)	80.60 [72.53–91.58)	< 0.0001*
Microvascular obstruction present, n (%)	37 (43)	127 (54)	0.078‡
Microvascular obstruction extent (%LV mass), mean±SD	1.96±3.95	3.25±5.32	0.039*
Myocardial haemorrhage, n (%)	17 (29)	85 (45)	**0.032‡**
T1 remote zone (ms), mean±SD	968.46±24.97	958.74±24.64	**0.003†**
T1 infarct zone, (ms), mean±SD	1105.40±52.39	1091.20±51.04	**0.042†**
T1 core zone, (ms), mean±SD	1001.05±74.43	995.68±51.18	0.615†
T2 remote zone (ms), mean±SD	50.07±2.11	49.62±2.04	0.088†
T2 infarct zone (ms), mean±SD	62.45±4.91	63.04±5.22	0.356†
T2 core zone (ms), mean±SD	53.10±4.68	54.15±4.87	0.204†
**MRI finding 6 months post-MI**			
Myocardial salvage index (%LV mass), median (IQR) Infarct size (%LV mass), median (IQR)	64.20 [47.55–87.15) 9.70 [3.45–17.63)	60.40 [43.80–82.30) 12.10 [4.40–19.90)	0.169* 0.156*
LVEF% at 6 months, mean±SD	63.93±9.13	61.15±9.41	**0.025†**
LVESVi at 6 months, mL/m^2^, median (IQR)	26.55 [18.70–33.20)	31.50 [22.68–40.93)	< 0.0001*
LVEDVi at 6 months, mL/m^2^, median (IQR)	74.25 [63.60–80.80)	83.70 [72.75–94.55)	< 0.0001*
Change in LVEDVi (6 months compared with 2 days), median (IQR)	0.90 [−7.10 to 7.40)	2.25 [−5.55 to 9.60)	0.158*
Change in LVEDVi (6 months compared with 2 days≥20%), n (%)	5 (6)	15 (7)	0.872‡
Persistent myocardial haemorrhage, n (%):			
None/resolved Persisting	43 (88) 6 (12)	121 (75) 41 (25)	0.054‡
T2 remote zone (ms), mean±SD	49.94±2.48	49.64±2.28	0.328†
T2 infarct zone (ms), mean±SD	56.41±3.95	56.05±4.24	0.512†
T2 core zone (ms), mean±SD	45.72±4.31	47.80±3.57	0.194†

LV volumes and ejection fraction on MRI at 2 days was available in 321 subjects. At 6 months, LV volumes were available in 294 subjects. Infarct size was available in 322 subjects on MRI at 2 days and 296 subjects at 6 months. Myocardial salvage index and LVEF at 6 months was available in 296 subjects. Presence/absence of microvascular obstruction was available in 324 subjects, extent of microvascular obstruction in 322 subjects, myocardial haemorrhage in 246 subjects and persistent myocardial haemorrhage in 211 subjects. T1 remote and infarct zone information was available in 288 subjects, and T1 core in 160 subjects. On MRI at 2 days, T2 remote/infarct zone information was available in 324 subjects, core zone in 192 subjects and at 6 months T2 remote, infarct, core zone was available in 297, 296 and 52 subjects, respectively.

*Mann-Whitney U test.

†Student’s t-test.

‡χ^2^ test.

LV, left ventricular; LVEDVi, LV end-diastolic volume index; LVEF, left ventricular ejection fraction; LVESVi, LV end-systolic volume index; MI, myocardial infarction; STEMI, ST-segment elevation myocardial infarction.

## Results

### Baseline characteristics

Of 372 patients with acute STEMI who were assessed for eligibility, 324 (87%) patients were enrolled. Reasons for non-enrolment are detailed in figure 1. There were no patients who presented with myocardial infarction with non-obstructive coronary arteries. Women accounted for 27% (n=87) of the cohort and had a mean age of 61±12.19 years. Twenty five (29%) women were young (age <55 years), which did not differ significantly from the proportion of men in the study with age <55 years (n=84; 35%), p=0.260. Primary PCI was performed in 302 (93%) subjects, the remainder had rescue PCI after failed thrombolysis (14; 4%) or PCI after successful thrombolysis (8; 2%)).

Baseline demographic and clinical features of the 324 patients with STEMI are shown in table 1. Women had higher baseline N-terminal pro-B-type natriuretic peptide levels, were less likely to have LAD territory infarctions and were less likely to have beta-blockers prescribed on discharge (all p<0.05). ECG, coronary physiology and angiographic characteristics are shown in table 2, and MRI findings are presented in table 3. The mean time between STEMI and baseline MRI was 2±1.90 days and did not differ between sexes (p=0.220).

### Acute reperfusion outcomes in relation to sex

Compared with men, women were less likely to have TIMI myocardial perfusion grade ≤1 (20% vs 32%, OR: 0.52, 95% CI: 0.28 to 0.97, p=0.040), after adjustment for estimated glomerular filtration rate (eGFR), reperfusion strategy (thrombolysis vs no thrombolysis), culprit artery territory (LAD/left main vs circumflex vs right coronary artery), age and ischaemic time. Consistent with this finding, female sex was a multivariable associate of lower corrected TIMI frame count (12.94 vs 17.65, β: −0.17, 95% CI: −0.28 to −0.05, p=0.004). There were no sex differences in incomplete ST-segment resolution on the ECG (OR: 0.89, p=0.743) (table 4). IMR and CFR measured directly in the culprit artery were similar in women and men (table 2).

**Table 4:** Univariable and multivariable associations between female sex and (I) MRI surrogate outcomes and (II) acute reperfusion outcomes from the ECG, angiogram and invasive coronary physiology

	Crude		Adjusted	
**MRI outcomes 2 days post-STEMI**	**β/OR (95% CI**)	**P value**	**β/OR (95% CI**)	**P value**
Infarct size (%LV mass)	−0.08 (−0.19 to 0.03)	0.140	−0.06 (−0.16 to 0.05)	0.282
LVEF%	0.10 (−0.01 to 0.21)	0.086	0.05 (−0.05 to 0.16)	0.327
LVESVi at 2 days, mL/m^2^	−0.22 (−0.33 to −0.12)	**< 0.0001**	−0.17 (−0.27 to −0.07)	**0.002**
LVEDVi at 2 days, mL/m^2^	−0.30 (−0.40 to −0.19)	**< 0.0001**	−0.26 (−0.36 to −0.20)	**< 0.0001**
Microvascular obstruction present	0.64 (0.39 to 1.05)	0.079	0.60 (0.35 to 1.02)	0.060
Microvascular obstruction extent (%LV mass)	−0.12 (−0.22 to −0.01)	**0.039**	−0.10 (−0.21 to −0.01)	0.077
Myocardial haemorrhage present	0.50 (0.27 to 0.95)	**0.033**	0.52 (0.26 to 1.03)	0.060
T1 remote zone (ms)	0.17 (0.06 to 0.29)	**0.003**	0.18 (0.06 to 0.30)	**0.004**
T1 infarct zone (ms)	0.12 (0.00 to 0.24)	**0.042**	0.11 (−0.01 to 0.23)	0.063
T1 core zone (ms)	0.04 (−0.12 to 0.20)	0.615	0.07 (−0.09 to 0.23)	0.398
T2 remote zone (ms)	0.10 (−0.01 to 0.20)	0.088	0.10 (−0.02 to 0.21)	0.089
T2 infarct zone (ms)	−0.05 (−0.16 to 0.06)	0.356	−0.06 (−0.18 to 0.06)	0.319
T2 core zone (ms)	−0.09 (−0.23 to 0.05)	0.204	−0.10 (−0.26 to 0.05)	0.176
**MRI outcomes 6 months post-STEMI**				
Myocardial salvage index (%LV mass)	0.08 (−0.03 to 0.20)	0.151	0.08 (−0.04 to 0.20)	0.206
Infarct size (%LV mass)	−0.10 (−0.22 to 0.01)	0.077	−0.07 (−0.18 to 0.04)	0.207
LVEF%	0.13 (0.02 to 0.25)	**0.025**	0.11 (0.00 to 0.23)	0.060
LVESVi at 6 months, mL/m^2^	−0.21 (−0.32 to −0.10)	**< 0.0001**	−0.19 (−0.31 to −0.08)	**0.001**
LVEDVi at 6 months, mL/m^2^	−0.27 (−0.38 to −0.16)	**< 0.0001**	−0.26 (−0.37 to −0.15)	**< 0.0001**
Change in LVEDVi>20%	0.92 (0.32 to 2.62)	0.872	−0.08 (−0.19 to 0.04)	0.206
Persistent myocardial haemorrhage present	0.48 (0.21 to 1.08)	0.075	0.47 (0.20 to 1.13)	0.092
T2 remote zone (ms)	0.06 (−0.06 to 0.17)	0.328	0.08 (−0.04 to 0.20)	0.193
T2 infarct zone (ms)	0.04 (−0.08 to 0.15)	0.512	0.04 (−0.08 to 0.16)	0.558
T2 core zone (ms)	−0.18 (−0.46 to 0.10)	0.194	0.01 (−0.40 to 0.42)	0.946
**Angiography outcomes**				
TIMI frame count post-PCI	−0.15 (−0.26 to −0.04)	**0.007**	−0.17 (−0.28 to −0.05)	**0.004**
TIMI myocardial perfusion grade post-PCI ≤1	0.51 (0.28 to 0.93)	**0.029**	0.52 (0.28 to 0.97)	**0.040**
**Invasive coronary physiology outcomes**				
IMR >40(U)	0.92 (0.51 to 1.64)	0.771	0.90 (0.49 to 1.66)	0.744
IMR(U)	−0.07 (−0.18 to 0.05)	0.247	−0.09 (−0.20 to 0.03)	0.161
CFR≤2	0.94 (0.47 to 1.89)	0.871	1.11 (0.54 to 2.30)	0.777
CFR	−0.03 (−0.14 to 0.09)	0.667	−0.00 (−0.13 to 0.12)	0.951
**ECG outcomes**				
Incomplete ST-segment resolution	0.89 (0.44 to 1.80)	0.743	1.17 (0.55 to 2.46)	0.687

MRI outcomes at 6 months were adjusted for prescription of beta-blocker on discharge, eGFR, reperfusion strategy (thrombolysis vs no thrombolysis), culprit artery territory (LAD/left main vs circumflex vs right coronary artery), age and ischaemic time. All other outcomes were adjusted for eGFR, reperfusion strategy (thrombolysis vs no thrombolysis), culprit artery territory (LAD/left main vs circumflex vs right coronary artery), age and ischaemic time.

CFR, coronary flow reserve; eGFR, estimated glomerular filtration rate; IMR, Index of microcirculatory resistance; LV, left ventricular; LVEDVi, LV end-diastolic volume index; LVEF, left ventricular ejection fraction; LVESVi, LV end-systolic volume index; PCI, percutaneous coronary intervention; STEMI, ST-segment elevation myocardial infarction; TIMI, Thrombolysis in Myocardial Infarction.

### Infarct pathology and sex-specific associations

#### Microvascular obstruction and myocardial haemorrhage

Microvascular obstruction revealed by MRI 2 days after reperfusion occurred in 164 subjects (70%) (table 3). The proportions of women and men affected by microvascular obstruction were similar (43% vs 54%, OR: 0.64, 95% CI: 0.39 to 1.05, p=0.079). In multivariable models including infarct territory (which differed significantly between sexes), the association between sex and the presence of microvascular obstruction remained non-significant (OR: 0.60, 95% CI: 0.35 to 1.02, p=0.060) (table 4).

Evaluable T2* maps on the baseline MRI were available in 246 patients (58 women, 67%; 188 men, 79%). Myocardial haemorrhage was present in 102 (41%) subjects, on baseline MRI, and occurred less frequently in women than men (29% vs 45%), but this difference was not significant after adjustment for eGFR, reperfusion strategy (thrombolysis vs no thrombolysis), culprit artery territory, age and ischaemic time (OR: 0.52, 95% CI: 0.26 to 1.03, p=0.060). Evaluable T2* maps on the 6-month MRI were available in 251 patients (61 women, 70%; 190 men, 55%). Persistent myocardial haemorrhage on the 6-month MRI occurred in 47 (22%) of subjects and was numerically lower in women (12% vs 25%), but this difference was not statistically significant (OR: 0.48, 95% CI: 0.21 to 1.08, p=0.075).

#### Remote zone inflammation

Female sex was a multivariable associate of higher T1 remote zone signal on baseline MRI (968.46 vs 958.74 ms, β: 0.18, 95% CI: 0.06 to 0.30, p=0.004). After adjustment for the same variables, T1 infarct zone signal was not associated with sex (β: 0.11, p=0.063). There were no sex differences in T1 core zone signal on baseline MRI, or T2 signal on MRI at baseline or at 6 months (table 4).

#### LV volumes and adverse remodelling

Adverse LV remodelling, defined as an increase in ≥20% LVEDV at 6 months from baseline, occurred in 6% of women and 7% of men (OR: 0.92, p=0.872). Women had smaller indexed LVEDVs than men 2 days (70.80 vs 80.60 mL/m^2^, β: −0.26, 95% CI: −0.36 to −0.20, p<0.0001) and 6 months post-MI (74.25 vs 83.70 mL/m^2^, β: −0.26, 95% CI: −0.37 to −0.15, p<0.0001). Women had smaller indexed LV end-systolic volumes than men 2 days (32.45 vs 36.30 mL/m^2^, β: −0.17, 95% CI: −0.27 to –0.07, p=0.002) and 6 months post-MI (26.55 vs 31.50 mL/m^2^, β: −0.19, 95% CI: −0.31 to −0.08, p<0.0001).

#### LVEF, Infarct size and myocardial salvage index

Infarct size, myocardial salvage and LV ejection fraction were similar in women and men at 2 days and 6 months post-MI (table 3).

### Health outcomes

**Figure 2:**
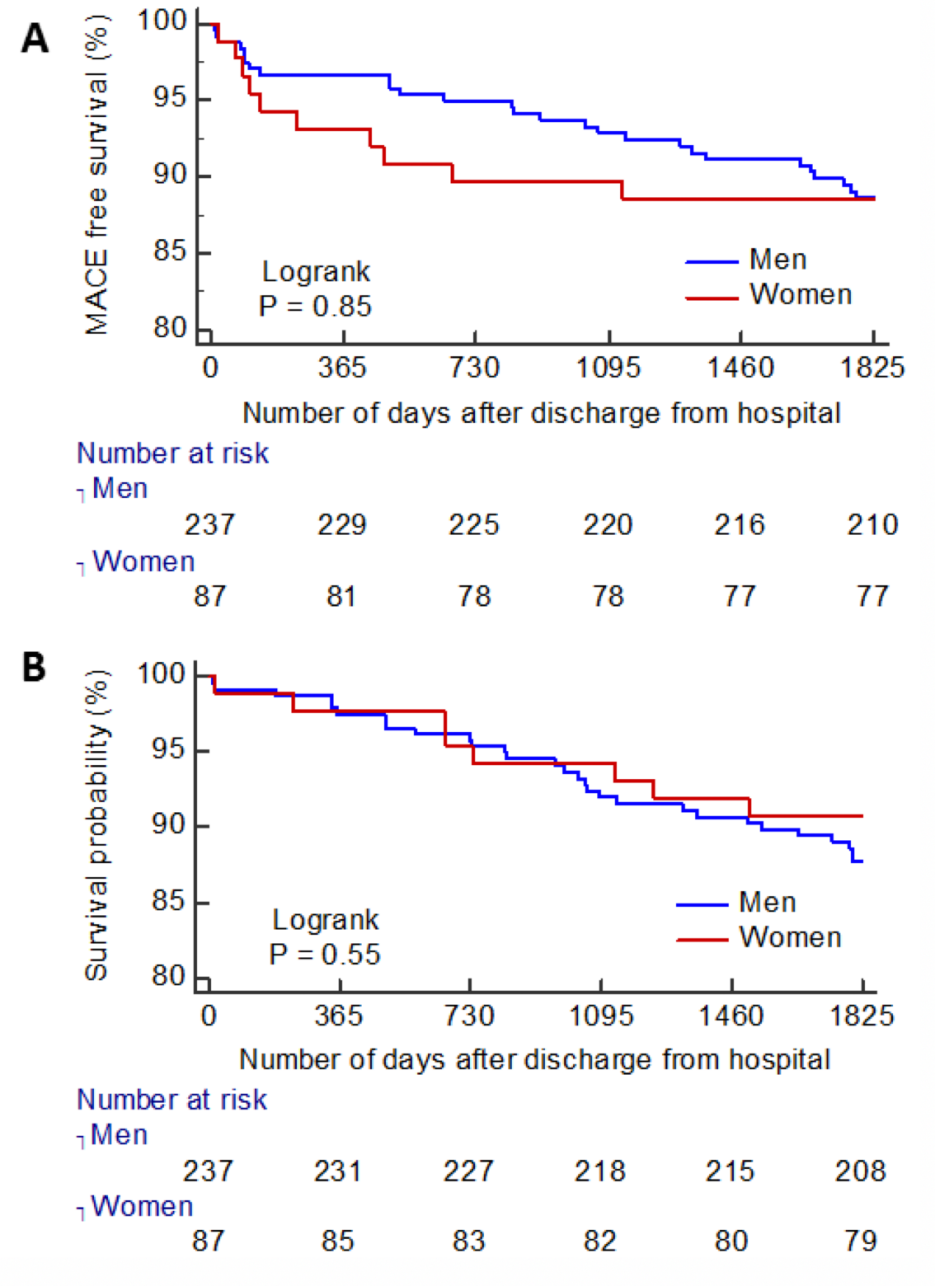
(A) Kaplan-Meier curve for MACE-free survival, after initial hospitalisation to 5-year follow-up. (B) Kaplan-Meier curve for survival free from all-cause death or the first heart failure hospitalisation to 5-year follow-up. MACE, major adverse cardiovascular event.

In the landmark survival analysis with median 5-year follow-up, female sex was not associated with either MACE (women n=10 [11.5%] vs men n=30 [14.5%], HR: 0.92, 95% CI: 0.45 to 1.89, p=0.820), or all-cause death or first hospitalisation for heart failure postdischarge (n=11 [13%] compared with men n=36 [15%], HR: 0.80, 95% CI: 0.41 to 1.57, p=0.520) (figure 2).

## Discussion

We have undertaken a large, contemporary, prospective study of sex-based associations in acute reperfusion injury, infarct characteristics and long-term prognosis after STEMI. Anterior MI was more common in men and beta-blockers were prescribed less often in women. We observed no sex differences in acute reperfusion injury assessed by IMR or CFR, but angiographically corrected TIMI frame count was lower and TIMI myocardial perfusion grade was higher in women than men. On MRI performed 2 days and 6 months later, there were no differences in microvascular obstruction, myocardial haemorrhage, infarct size, myocardial salvage index, LV remodelling or LV function. 3 6Long-term health outcomes in women and men were similar.

### Microvascular reperfusion injury in women post-STEMI

The angiographic finding of lower corrected TIMI frame count and higher myocardial perfusion grade in women was a surprise finding, and perhaps suggests that PCI was less effective in women than men. Prior studies have reported that women were more likely to have pre-infarct angina than men^4 5^ implicating ischaemic preconditioning as a plausible explanation.^21^ The comparatively low rates of pre-infarct angina, without sex differences (women 10% vs males 13%; p=0.507), do not implicate this explanation in our cohort. We considered whether sex differences in lesion plaque characteristics at the time of angiography may have contributed to the lower TIMI myocardial perfusion grade and higher corrected TIMI frame count in men. However, no sex differences were detected in culprit lesion plaque characterisation score, or total number of complex plaques (table 2). We also found no sex differences in intraprocedural thrombotic events, which is consistent with previous reports.^17^ In a previous study, male patients with STEMI had higher plaque burden than propensity-matched women, as revealed by virtual histology intravascular ultrasound^22^; however high-risk lesion characteristics such as thin cap fibroatheroma were similar in both sexes.^22^

Even though we observed angiographically lower corrected TIMI frame counts and higher TIMI myocardial perfusion grades at the end of the PCI procedure in women than men, there were no sex differences in invasive coronary physiology measurements of acute microvascular reperfusion injury, that is, IMR and CFR. We considered whether this result could be explained by men in our cohort having more LAD territory infarcts. However, even when the analysis was restricted to patients with IMR measured in the LAD, there was still no sex difference in IMR.

The inconsistency between the acute angiographic and invasive coronary physiology observations might be explained by angiography-based techniques for assessing microvascular function having lower specificity and poorer interobserver and intraobserver reproducibility, largely related to dependence on volume of contrast dye injected or force of injection, coronary artery size and haemodynamic conditions. Even though corrected TIMI frame count measured in the LAD was divided by 1.7 to correct for the relatively longer vessel length, it is plausible that the higher proportion of LAD infarcts in males may still have contributed to the higher corrected TIMI frame count in males. IMR has previously been shown to have better predictive utility for microvascular obstruction than angiographic parameters.^23^ The IMR and CFR results were consistent with the MRI findings. Furthermore, the CIs are narrow for the non-significant associations between sex and the presence of microvascular obstruction or myocardial haemorrhage, which suggests that type II statistical error was unlikely (table 4).

Microvascular reperfusion plays an important role in ameliorating adverse LV remodelling^24^ and prior literature suggests that LV remodelling is more favourable in women, potentially due to sex differences in modulation of the ischaemia-induced apoptotic cascade.^25^ In our cohort, LV remodelling was not significantly different between men and women. Furthermore, N-terminal pro-B-type natriuretic peptide level at 6 months (a biomarker of adverse LV remodelling^19^) did not differ between women and men. We found that myocardial remote zone native T1 (a biomarker of inflammation and risk factor for adverse LV remodelling^19^) was higher in women. However, there were no sex differences in remote zone native T2. Taken together, the findings suggest that the sex differences in remote zone T1 may be explained by women generally having higher myocardial T1 values than men^26^ and not due to a higher degree of inflammation of the remote zone in women.

Our data contrast with those of Canali *et al*,^4^ who reported that women had more favourable microvascular obstruction extent, infarct size and myocardial salvage post-STEMI. Our study extends the findings of Canali *et al*^4^ by providing new information on coronary physiology acutely, myocardial haemorrhage at 2 days and 6 months, and long-term clinical outcomes.

#### In-hospital acute MI care and beta-blocker therapy

The door-to-balloon time, a measure of the efficiency of emergency in-hospital care for STEMI, was short and similar in women and men. Furthermore, in contrast to previous studies, ischaemic time (which is causally implicated in the development of microvascular obstruction) was similar in women and men in our contemporary cohort. Other in-hospital treatments were also similar. On the other hand, beta-blocker therapy was prescribed less often in women at discharge. The reasons for this sex-based difference are unclear. Beta-blocker therapy notably with metoprolol may reduce reperfusion injury and improve prognosis (including in women),^27^ and beta-blocker therapy post-MI is recommended in practice guidelines.^28^ Previous studies have found that women were less likely than men to adhere effectively to chronic medications including after MI.^29^ The difference in beta-blocker therapy serves another reminder of sex differences in post-MI care in contemporary practice.

### Limitations

This is an observational study, so causality cannot be inferred. The data are from a single centre, but it is still likely to be representative of contemporary patients with STEMI in other centres. The study was not powered to demonstrate sex differences in clinical outcomes. We do not have information on medication adherence postdischarge and do not have information on the reason for beta-blockers being prescribed less often in women.

## Conclusions

There were no sex differences in serial measures of microvascular reperfusion injury from acute invasive coronary physiology and MRI performed 2 days and 6 months later. LV remodelling and health outcomes were also similar. Beta-blocker therapy at discharge was prescribed less often in women than men, and anterior MI was more common in men.

10.1136/openhrt-2018-000979.supp1 Supplementary data
