# Supplementary material for: Sex-based associations with microvascular injury and outcomes after ST-segment elevation myocardial infarction
Source: Open Heart. 2019 Apr 29;6(1):e000979. doi: 10.1136/openhrt-2018-000979 (PMC6519583; doi:10.1136/openhrt-2018-000979)
Supplement: Supplementary data [file openhrt-2018-000979supp001.docx]

# SUPPLEMENTAL METHODS

# Sex-Based Associations with Microvascular Injury and Outcomes after ST-Segment Elevation Myocardial Infarction

# ClinicalTrials.gov identifier: NCT02072850.Table of contents

Setting and study populations…………………………………………………………………3

Percutaneous coronary intervention…………………………………………………………...3

Measurement of IMR and CFR at the end of PCI……………………………………………..4

Angiographic analysis…………………………………………………………………………4

MRI acquisition and analyses…………………………………………………………………5

Electrocardiogram……………………………………………………………………………..9

Biochemical and haematological measurements……………………………………………..10

Statistics……………………………………………………………………………………...11

References……………………………………………………………………………………13

# Setting and study populations

### STEMI patients

Screening, enrolment, and data collection were performed prospectively by cardiologists in the cardiac catheterisation laboratories of the Golden Jubilee National Hospital, Glasgow, United Kingdom - a regional referral centre for percutaneous coronary intervention (PCI). The hospital provides clinical services for a population of 2.2 million. A screening log was recorded, including patients who did not participate in the cohort study. Patients were invited to undergo cardiac magnetic resonance imaging (MRI) 2 days and 6 months after hospital admission (1)(2).

# Percutaneous coronary intervention

Consecutive acute STEMI patients were screened for the inclusion and exclusion criteria. During ambulance transfer to the hospital, the patients received 300 mg of aspirin, 600 mg of clopidogrel and 5000 IU of unfractionated heparin (3, 4). The initial primary PCI procedure was performed using radial artery access. Conventional bare metal and drug eluting stents were used in accordance with guideline recommendations and clinical judgement. During PCI, glycoprotein IIbIIIa inhibitor therapy was initiated with high dose tirofiban (25μg/kg/bolus) followed by an intravenous infusion of 0.15μg/kg/min for 12 hours, according to clinical judgement and indications for bail-out therapy (3, 4). No reflow was treated according to contemporary standards of care with intra-coronary nitrate (i.e. 200μg) and adenosine (i.e. 30 – 60μg) (3, 4), as clinically appropriate.

# Measurement of IMR and CFR at the end of PCI

A dual sensor pressure- and temperature-sensitive coronary guidewire (Abbott Vascular, Santa Clara, California) was used to measure coronary flow reserve (CFR) and the index of microcirculatory resistance (IMR) in the culprit coronary artery at the end of primary or rescue PCI. The guidewire was calibrated outside the body, equalised with aortic pressure at the ostium of the guide catheter and then advanced to the distal third of the culprit artery.

CFR is defined as the mean transit time at rest divided by the mean transit time during hyperaemia. IMR is defined as the distal coronary pressure multiplied by the mean transit time of a 3ml bolus of saline at room temperature during maximal coronary hyperaemia, measured simultaneously (mmHg x s, or units) (5-6). We have previously assessed the repeatability of IMR using duplicate measurements 5 minutes apart in a subset of 12 consecutive patients (7).

# Angiographic analysis

### **Myocardial perfusion grade**

Angiographic evidence of myocardial perfusion was evaluated using the TIMI myocardial perfusion grade at the end of the PCI procedure (8).

| Grade |  |
| --- | --- |
| 0 | No myocardial blush |
| 1 | Minimal blush and very slow clearing (e.g. present at beginning of next cine) |
| 2 | Good blush with slow clearing of myocardial contrast (present at end of cine but gone at beginning of next) |
| 3 | Good blush and normal clearing (ie. gone by end of cine) |

**Assessment by corrected TIMI Frame Count**

Corrected TIMI frame count (cTFC) was calculated as the number of frames for dye to reach a standardised distal landmark in each angiographic territory. The first frame taken for the measurement was the frame in which dye touched both borders of the coronary artery in question and moved forward with at least 70% of the vessel lumen opacified. The standardised distal landmarks were taken as the first branch of the postero-lateral artery for the right coronary artery, the most distal branch of the obtuse marginal for the circumflex, and the distal bifurcation of the left anterior descending (LAD) coronary artery. The number of frames from the first frame to the last frame when the dye entered the standardised distal landmark was counted. To correct for the relatively increased length of the LAD, the cTFC in the LAD was divided by 1.7.

# MRI acquisition and analyses

## MRI acquisition

MRI was performed on a Siemens MAGNETOM Avanto (Erlangen, Germany) 1.5-Tesla scanner with a 12-element phased array cardiac surface coil. T2 maps were acquired in contiguous short axis slices covering the whole ventricle, using an investigational prototype T2-prepared (T2P) TrueFisp sequence (9, 10). Typical imaging parameters were: bandwidth ~947 Hz/pixel; flip angle 70°; T2 preparations: 0ms, 24ms, and 55ms respectively; matrix 160 x 105 pixels; spatial resolution 2.6 x 2.1 x 8.0 mm; slice thickness 8mm.

T2*-maps were obtained using an investigational prototype T2* map sequence acquired in 3 short-axis slices (basal, mid and apical). Typical imaging parameters were: bandwidth ~814 (x8) Hz/pixel; flip angle 18°; matrix 256x115; spatial resolution 2.6 x 1.6 x 10 mm; slice thickness 8 mm.

To assess early microvascular obstruction, early gadolinium enhancement imaging was acquired 1, 3, 5 and 7 minutes post-contrast injection using a TrueFISP readout and fixed inversion time (TI) of 440ms. Late gadolinium enhancement images covering the entire LV were acquired 10-15 minutes after intravenous injection of 0.15 mmol/kg of gadoterate meglumine (Gd^2+^-DOTA, Dotarem, Guebert S.A.) using segmented phase-sensitive inversion recovery (PSIR) turbo fast low-angle shot (11). Microvascular obstruction was defined as a dark zone on early delayed enhancement imaging 1, 3, 5 and 7 minutes post-contrast injection and within an area of late gadolinium enhancement. Typical imaging parameters were: matrix = 192 x 256, flip angle = 25°, TE = 3.36ms, bandwidth = 130 Hz/pixel, echo spacing = 8.7ms and trigger pulse = 2. The voxel size was 1.8 x 1.3 x 8 mm^3^. Inversion times were individually adjusted to optimise nulling of apparently normal myocardium (typical values, 200 to 300ms).

## MR image analyses

The images were analysed on a Siemens work-station by observers with at least 3 years MRI experience. All of the images were reviewed by experienced MRI cardiologists. LV dimensions, volumes and ejection fraction were quantified using computer assisted planimetry (syngo MR®, Siemens Healthcare, Erlangen, Germany). All scan acquisitions were spatially co-registered.

*T2 and T2* – standardised measurements in myocardial regions of interest*

LV contours were delineated with computer assisted planimetry on the raw T2* image and the last corresponding T2 raw image, with echo time of 55ms (12). Contours were then copied onto the colour-encoded spatially co-registered maps and corrected when necessary by consulting the SSFP cine images. Apical segments were not included because of partial volume effects. Particular care was taken to delineate regions of interest with adequate margins of separation from tissue interfaces prone to partial volume averaging such as between myocardium and blood. Each T2/ T2* map image was visually assessed for the presence of artefacts relating to susceptibility effects or cardio-respiratory motion. Each map was evaluated against the original images. When artefacts occurred, the affected segments were not included in the analysis.

T2/ T2* values were segmented spatially and regions of interest were defined as (i) remote myocardium, (ii) injured myocardium and (iii) infarct core. The regions-of-interest were planimetered to include the entire area of interest with distinct margins of separation from tissue interfaces to exclude partial volume averaging. The remote myocardial region-of-interest was defined as myocardium 180º from the affected zone with no visible evidence of infarction, oedema or wall motion abnormalities (assessed by inspecting corresponding contrast enhanced T1-weighted, T2-weighted and cine images, respectively). The infarct zone region-of-interest was defined as myocardium with pixel values (T2) >2 SD from remote myocardium on T2-weighted MRI (10, 11). The infarct core was defined as an area in the centre of the infarct territory having a mean T2/ T2* value of at least 2 standard deviations (SDs) below the T2/ T2* value of the periphery of the area-at-risk.

In healthy volunteers, the mid-ventricular T2/T2* map was segmented into 6 equal segments, using the anterior right ventricular-LV insertion point as the reference point. T2/T2* was measured in each of these segments, and regions-of-interest were planimetered distinct and separate from blood-pool and tissue interfaces. These segmental values were also averaged to provide one value per subject. Results are presented as average values for segments and slices.

### Infarct definition and size

The presence of acute infarction was established based on abnormalities in cine wall motion, rest first-pass myocardial perfusion, and delayed-enhancement imaging. In addition, supporting changes on the ECG and coronary angiogram were also required. Acute infarction was considered present only if late gadolinium enhancement was confirmed on both the axial and long axis acquisitions. The myocardial mass of late gadolinium (grams) was quantified using computer assisted planimetry and the territory of infarction was delineated using a signal intensity threshold of >5 standard deviations above a remote reference region and expressed as a percentage of total LV mass. Infarct regions with evidence of microvascular obstruction were included within the infarct area and the area of microvascular obstruction was assessed separately and also expressed as a percentage of total LV mass.

### Microvascular obstruction

Microvascular obstruction was defined as a dark zone on EGE imaging 1, 3, 5 and 7 minutes post-contrast injection that remained present within an area of late gadolinium enhancement at 15 minutes.

### Myocardial haemorrhage

Myocardial haemorrhage was scored visually. On the T2* maps, a region of reduced signal intensity within the infarcted area, with a T2* value of <20ms (12-15), was considered to confirm the presence of myocardial haemorrhage.

### Myocardial oedema

The extent of myocardial oedema was defined as LV myocardium with pixel values (T1/T2) >2 standard deviations from remote myocardium.

### Myocardial salvage

Myocardial salvage was calculated by subtraction of percent infarct size from percent area-at-risk. The myocardial salvage index was calculated by dividing the myocardial salvage area by the initial area-at-risk (16-19).

### Adverse remodelling

Adverse remodelling was defined as an increase in LV end-diastolic volume ≥ 20% at 6 months from baseline (20).

### Reference ranges

Reference ranges used in the laboratory were 105 – 215 g for LV mass in men, 70 – 170 g for LV mass in women, 77 – 195 ml for LV end-diastolic volume in men, 52 – 141 ml for LV end-diastolic volume in women, 19 – 72 ml for LV end-systolic volume in men and 13 – 51 ml for LV end-systolic volume in women.

# Electrocardiogram

A 12-lead electrocardiogram (ECG) was obtained before coronary reperfusion and 60 minutes afterwards with Mac-Lab® technology (GE Healthcare) in the catheter laboratory and a MAC 5500 HD recorder (GE Healthcare) in the Coronary Care Unit. The ECGs were acquired by trained cardiology staff. The ECGs were de-identified and transferred to the local ECG management system. The ECGs were then analysed by the University of Glasgow ECG Core Laboratory which is certified to ISO 9001: 2008 standards as a UKAS Accredited Organization.

The extent of ST-segment resolution on the ECG assessed 60 minutes after reperfusion compared to the baseline ECG before reperfusion was expressed as complete (≥70%), incomplete (30% to <70%) or none (≤30%). ECG evidence of reperfusion injury was taken as persistence of ST segment elevation resolution post-procedure, and specifically ≤30% ST-segment resolution post-PCI.

# Biochemical and hematologic measurements

Blood samples were obtained immediately after reperfusion in the cardiac catheterization laboratory, and subsequently between 0600 - 0700 hrs each day during the initial in-patient stay in the Coronary Care Unit.

## Biochemical assessment of infarct size

Troponin T was measured (Elecsys Troponin T, Roche) as a biochemical measure of infarct size. The high sensitive assay reaches a level of detection of 5 pg/ml and achieves less than 10% variation at 14 pg/ml corresponding to the 99th percentile of a reference population. The peak troponin T value for each patient was recorded in the study database.

## Biochemical assessment of inflammation

C-reactive protein (CRP) was measured in an NHS hospital biochemistry laboratory using a particle enhanced immunoturbimetric assay method (Cobras C501, Roche) and the manufacturers calibrators and quality control material, as a biochemical measure of inflammation. The high sensitive assay CRP measuring range is 0.1-250 mg/L. The expected CRP values in a healthy adult are < 5 mg/L, and the reference range in our hospital is 0 - 10 mg/L.

## Pre-specified health outcomes

We pre-specified adverse health outcomes that are pathophysiologically linked with STEMI. The primary composite outcome was all-cause death or first heart failure hospitalization following the initial admission.

Research staff screened for events from enrolment by checking the medical records and by contacting patients and their primary and secondary care physicians, as appropriate with no loss to follow-up. Each serious adverse event (SAE) was reviewed by a cardiologist who was independent of the research team and blinded to all of the clinical and MRI- data. The SAEs were defined according to standard guidelines and categorised as having occurred either during the index admission or post-discharge.

Clinical events were assessed and validated by an independent cardiologist who had access to relevant source clinical data. This cardiologist followed an agreed charter and he was blinded to all of the other clinical data.

# Statistics

### Sample size calculation for the whole cohort

With an estimated haemorrhage incidence of 33% at 48 h post-STEMI, 100 subjects would have evidence of myocardial haemorrhage and 200 subjects would not. The study would have 90% power at a 5% level of significance using a two-sided two sample t-test to detect a between-group difference in a baseline variable of interest e.g. index of microcirculatory resistance equivalent to three eighths of a common standard deviation. We also estimated that at least 30 major adverse cardiac events (MACE) would occur based on a conservative estimate of the event rate (10-12%) at 18 months. The sample size calculation was performed using nQuery version 7.0.

### Inter-rater reliability

Two raters assessed the angiograms of 30 subjects randomly selected from the whole cohort. Inter-rater reliability for angiographic parameters was assessed using weighted Cohen’s kappa and the intra-class correlation coefficient (ICC) with random effects models.

Trial Management

The study was conducted in line with Guidelines for Good Clinical Practice (GCP) in Clinical Trials. <http://www.mrc.ac.uk/documents/pdf/good-clinical-practice-in-clinical-trials/>

Trial management included a Trial Management Group, and an independent Clinical Trials Unit. Day to day study activity was coordinated by the Trial Management Group who was responsible to the Sponsor which was responsible for overall governance and that the trial was conducted according to GCP standards.

# References

1. Kramer CM, Barkhausen J, Flamm SD, Kim RJ, Nagel E. Standardised cardiovascular magnetic resonance (CMR) protocols 2013 update. *J Cardiovasc Magn Reson.* 2013; 15: 91.

2. Cerqueira MD, Weissman NJ, Dilsizian V, Jacobs AK, Kaul S, Laskey WK, Pennell DJ, Rumberger JA, Ryan T, Verani MS; American Heart Association Writing Group on Myocardial Segmentation and Registration for Cardiac Imaging. Standardised myocardial segmentation and nomenclature for tomographic imaging of the heart. A statement for healthcare professionals from the Cardiac Imaging Committee of the Council on Clinical Cardiology of the American Heart Association. *Circulation*. 2002; 105: 539-542.

3. Task Force on the management of ST-segment elevation acute myocardial infarction of the European Society of Cardiology (ESC), Steg PG, James SK, Atar D, Badano LP, Blömstrom-Lundqvist C, Borger MA, Di Mario C, Dickstein K, Ducrocq G, Fernandez-Aviles F, Gershlick AH, Giannuzzi P, Halvorsen S, Huber K, Juni P, Kastrati A, Knuuti J, Lenzen MJ, Mahaffey KW, Valgimigli M, van 't Hof A, Widimsky P, Zahger D. ESC Guidelines for the management of acute myocardial infarction in patients presenting with ST-segment elevation. *Eur Heart J*. 2012; 33: 2569-2619.

4. Windecker S, Kolh P, Alfonso F, Collet JP, Cremer J, Falk V, Filippatos G, Hamm C, Head SJ, Juni P, Kappetein AP, Kastrati A, Knuuti J, Landmesser U, Laufer G, Neumann FJ, Richter DJ, Schauerte P, Sousa Uva M, Stefanini GG, Taggart DP, Torracca L, Valgimigli M, Wijns W, Witkowski A. 2014 ESC/EACTS Guidelines on myocardial revascularization: The Task Force on Myocardial Revascularization of the European Society of Cardiology (ESC) and the European Association for Cardio-Thoracic Surgery (EACTS)Developed with the special contribution of the European Association of Percutaneous Cardiovascular Interventions (EAPCI). *Eur Heart J.* 2014; 35: 2541-2619.

5. Fearon WF, Shah M, Ng M, Brinton T, Wilson A, Tremmel JA, Schnittger I, Lee DP, Vagelos RH, Fitzgerald PJ, Yock PG, Yeung AC. Predictive value of the index of microcirculatory resistance in patients with ST-segment elevation myocardial infarction. *J Am Coll Cardiol.* 2008; 51: 560-565.

6. McGeoch R, Watkins S, Berry C, Steedman T, Davie A, Byrne J, Hillis S, Lindsay M, Robb S, Dargie H, Oldroyd K. The index of microcirculatory resistance measured acutely predicts the extent and severity of myocardial infarction in patients with ST-segment elevation myocardial infarction. *JACC Cardiovasc Interv.* 2010; 3: 715-722.

7. Payne AR, Berry C, Doolin O, McEntegart M, Petrie MC, Lindsay MM, Hood S, Carrick D, Tzemos N, Weale P, McComb C, Foster J, Ford I, Oldroyd KG. Microvascular Resistance Predicts Myocardial Salvage and Infarct Characteristics in ST-Elevation Myocardial Infarction. *J Am Heart Assoc. 2012; 1: e002246.*

8. Gibson CM, Karha J, Giugliano RP, Roe MT, Murphy SA, Harrington RA, Green CL, Schweiger MJ, Miklin JS, Baran KW, Palmeri S, Braunwald E, Krucoff MW; INTEGRITI Study Group. Association of the timing of ST-segment resolution with TIMI myocardial perfusion grade in acute myocardial infarction. *Am Heart J.* 2004; 147: 847-852.

9. Giri S, Chung YC, Merchant A, Mihai G, Rajagopalan S, Raman SV, Simonetti OP. T2 quantification for improved detection of myocardial edema. *J Cardiovasc Magn Reson.* 2009; 11: 56.

10. Verhaert D, Thavendiranathan P, Giri S, Mihai G, Rajagopalan S, Simonetti OP, Raman SV. Direct T2 quantification of myocardial edema in acute ischemic injury. *JACC Cardiovasc Imaging.* 2011; 4: 269-278.

11. Kellman P, Arai AE, McVeigh ER, Aletras AH. Phase-sensitive inversion recovery for detecting myocardial infarction using gadolinium-delayed hyperenhancement. *Magn Reson Med.* 2002; 47: 372-383.

12. Ghugre NR, Ramanan V, Pop M, Yang Y, Barry J, Qiang B, Connelly KA, Dick AJ, Wright GA. Quantitative tracking of edema, haemorrhage, and microvascular obstruction in subacute myocardial infarction in a porcine model by MRI. *Magn Reson Med*. 2011; 66: 1129-1141.

13. Kandler D, Lucke C, Grothoff M, Andres C, Lehmkuhl L, Nitzsche S, Riese F, Mende M, de Waha S, Desch S, Lurz P, Eitel I, Gutberlet M. The relation between hypointense core, microvascular obstruction and intramyocardial haemorrhage in acute reperfused myocardial infarction assessed by cardiac magnetic resonance imaging. *Eur Radiol.* 2014; 24: 3277-3288.

14. O'Regan DP, Ariff B, Neuwirth C, Tan Y, Durighel G, Cook SA. Assessment of severe reperfusion injury with T2* cardiac MRI in patients with acute myocardial infarction. *Heart.* 2010; 96: 1885-1891.

15. Anderson LJ, Holden S, Davis B, Prescott E, Charrier CC, Bunce NH, Firmin DN, Wonke B, Porter J, Walker JM, Pennell DJ. Cardiovascular T2-star (T2*) magnetic resonance for the early diagnosis of myocardial iron overload. *Eur Heart J.* 2001; 22: 2171-2179.

16. Eitel I, Desch S, Fuernau G, Hildebrand L, Gutberlet M, Schuler G, Thiele H. Prognostic significance and determinants of myocardial salvage assessed by cardiovascular magnetic resonance in acute reperfused myocardial infarction. *J Am Coll Cardiol.* 2010; 55: 2470-2479.

17. Berry C, Kellman P, Mancini C, Chen MY, Bandettini WP, Lowrey T, Hsu LY, Aletras AH, Arai AE. Magnetic resonance imaging delineates the ischemic area at risk and myocardial salvage in patients with acute myocardial infarction. *Circ Cardiovasc Imaging.* 2010; 3: 527-535.

18. Payne AR, Casey M, McClure J, McGeoch R, Murphy A, Woodward R, Saul A, Bi X, Zuehlsdorff S, Oldroyd KG, Tzemos N, Berry C. Bright-blood T2-weighted MRI has higher diagnostic accuracy than dark-blood short tau inversion recovery MRI for detection of acute myocardial infarction and for assessment of the ischemic area at risk and myocardial salvage. *Circ Cardiovasc Imaging.*2 011; 4: 210-219.

19. Francone M, Bucciarelli-Ducci C, Carbone I, Canali E, Scardala R, Calabrese FA, Sardella G, Mancone M, Catalano C, Fedele F, Passariello R, Bogaert J, Agati L. Impact of primary coronary angioplasty delay on myocardial salvage, infarct size, and microvascular damage in patients with ST-segment elevation myocardial infarction: insight from cardiovascular magnetic resonance. *J Am Coll Cardiol.* 2009; 54: 2145-2153.

20. Carrick D, Haig C, Rauhalammi S, Ahmed N, Mordi I, McEntegart M, Petrie MC, Eteiba H, Lindsay M, Watkins S, Hood S, Davie A, Mahrous A, Sattar N, Welsh P, Tzemos N, Radjenovic A, Ford I, Oldroyd KG, Berry C. Pathophysiology of LV Remodeling in Survivors of STEMI: Inflammation, Remote Myocardium, and Prognosis. *JACC Cardiovasc Imaging.* 2015; 8: 779-789.
